# Optimized extraction of polyphenolic antioxidants from the leaves of Himalayan Oak species

**DOI:** 10.1371/journal.pone.0259350

**Published:** 2021-11-03

**Authors:** Aseesh Pandey, Tarun Belwal, Sushma Tamta, Ranbeer S. Rawal

**Affiliations:** 1 G.B. Pant ‘National Institute of Himalayan Environment’ (NIHE), Almora, Uttarakhand, India; 2 G.B. Pant ‘National Institute of Himalayan Environment’ (NIHE), Sikkim Regional Centre, Gangtok, East Sikkim, India; 3 Department of Botany, D.S.B. Campus, Kumaun University, Nainital, Uttarakhand, India; Universite d’Orleans, FRANCE

## Abstract

In this study heat-assisted extraction conditions were optimized to enhance extraction yield of antioxidant polyphenols from leaves of Himalayan *Quercus* species. In initial experiments, a five-factor Plackett-Burman design including 12 experimental runs was tested against the total polyphenolic content (TP). Amongst, X_A_: extraction temperature, X_C_: solvent concentration and X_E_: sample-to-solvent ratio had shown significant influence on yield. These influential factors were further subject to a three-factor-three-level Box-Wilson Central Composite Design; including 20 experimental runs and 3D response surface methodology plots were used to determine optimum conditions [i.e. X_A_: (80°C), X_C_:(87%), X_E_: (1g/40ml)].This optimized condition was further used in other *Quercus* species of western Himalaya, India. The High-Performance Liquid Chromatography (HPLC) revealed occurrence of 12 polyphenols in six screened *Quercus* species with the highest concentration of catechin followed by gallic acid. Amongest, *Q*. *franchetii* and *Q*. *serrata* shared maximum numbers of polyphenolic antioxidants (8 in each). This optimized extraction condition of *Quercus* species can be utilized for precise quantification of polyphenols and their use in pharmaceutical industries as a potential substitute of synthetic polyphenols.

## 1. Introduction

The health benefits of polyphenols to human beings have been attracted attention to them worldwide and these beneficial properties of polyphenols are generally accredited to their antioxidant nature [1]. Polyphenolic antioxidants (PA) are structural class of organic chemicals which contain phenol units and based on their origin PAs can be classified in two categories i) synthetic and ii) natural. Structurally, these are classified into i) Phenolic acids ii) Flavonoids iii) Lignans and iv) Stilbenes. Synthetic PAs such as butylated hydroxyanisole (BHA), butylated hydroxytoluene (BHT), propyl gallate (PG), tertiary-butylhydroquinone (TBHQ), octyl gallate (OG), dodecyl gallate (DG) etc. have been used in product formulations since 1950s in order to prevent or delay the onset of lipid oxidation during processing and storage of fats, oils and lipid containing foods [2]. Although these are effective in numerous food systems; yet their use in the food industry is declining because of safety concerns and reports regarding the toxic effects of synthetic polyphenols are available [3, 4]. Amongst, BHA and BHT have observed susceptive in causing liver damage and carcinogenesis; also, BHA, TBHQ, PG are reported to cause damage in double helical structure of DNA [3, 5]. This has led interest of consumers towards natural products and the market for natural antioxidants is estimated 50% larger than it is for synthetics [6].

Naturally, in plants, PAs are among the most abundant secondary metabolites with approximately 8,000 known structures [7–10]. These are used in cosmetics and nutraceutical industries [11, 12] and due to their diverse uses and superiority over synthetic PAs, nowadays, studies are focusing on identifying natural sources of PAs which are low-cost and abundant in nature [13, 14].

The *Quercus* genus (family: Fagaceae) consists 500 species of trees and shrubs distributed mainly in Central America and Southeast Asia [15]. In the Himalayas, *Quercus* forms gregarious forest patches and sometimes grows in mixed forest formations with other broadleaved tree species, particularly in transition zones along the elevational gradient. *Quercus* species are rich in polyphenols and well-studied for their antiseptic, antidiarrheal, antimicrobial, anti-inflammatory, antioxidant, antitumoral, cytotoxic, gastroprotective, hemostatic, stomachic agent, and wound healing properties [16–18]. Leaves of *Quercus* species such as *Q*. *resinosa*, *Q*. *laeta*, *Q*. *grisea*, *Q*. *obtusata*, *Q*. *robur*, *Q*. *serrata* etc. are reported to have antioxidant and antimicrobial activity [19–22].

In plants, PAs occur in variable polarity states because of their diverse structural and physicochemical properties [23]. In food systems different interactions in between phenols and phenols with other constituents like acids, alkyl groups, sugars, etc. are reported emanating complex phenolic compounds such as polymeric phenols, condensed tannins, etc. [24]. Several methods such as enzymatic treatments, far-infrared radiation and heat treatments are known for the extraction of natural antioxidant polyphenols [25–28]. However, due to the variety in polyphenols and diverse nature of PAs, no single extraction technique is ideal for their optimum extraction [29, 30]. Traditional extraction methods used in extraction of PAs are energy and time consuming, also require larger solvent quantity that in some cases causes toxicity [31]. However, with the technological development, advanced extraction techniques and methods have been emerged for the improved and low-cost recovery of valuable PAs with comparatively less use of solvents, time, and energy [32, 33].

Optimization of extraction conditions is essential for economic commercial extraction process and can be achieved by considering several factors using empirical and/or statistical methods. Optimization factors, viz. solvent type and concentration, sample-to-solvent ratio, extraction time and extraction temperature etc., are known to influence the yield and quality of the desired compounds, and avoids chemical modifications [20, 34–36]. Response surface method (RSM) is reported effective in optimizing extraction procedures by investigating several influencing factors to the response and their interactions at one time with less experimental runs [37]. In recent studies, RSM has been used to optimize multifactor extraction conditions for polyphenolic compounds [19, 31, 37–40]. Based on the reviewed literatures, no optimal conditions are available for the extraction of PAs from Himalayan oak species. Thus, the present study envisaged to achieve optimum yield of PAs from leaves of Himalayan *Quercus* species through optimized heat assisted extraction (HAE) conditions.

## 2. Materials and methods

### 2.1. Plant material

In the month of June leaves from six Himalayan *Quercus* (oak) species viz. *Q*. *floribunda* Lindl. ex A. Camus (tilonj), *Q*. *franchetii* Skan (rianj), *Q*. *glauca* Thunb. (falant), *Q*. *serrata* Murrary (tasar oak), *Q*. *oblongata* D. Don (banj) and *Q*. *semecarpifolia* Sm (kharsu) were collected from Uttarakhand, West Himalaya, India. Leaves were dried at room temperature until the constant weight achieved in successive weighing. Dried leaves were milled and stored at −20°C in separately sealed plastic bags; till further analysis.

Species identity was confirmed by consulting the available herbarium records of *Quercus* species at the departmental herbaria and authenticated by departmental taxonomist. The nomenclature of *Quercus* species follows the online source of The Plant List (http://www.theplantlist.org). The details of target species have been provided in S1 Table in S1 File.

### 2.2. Chemical and reagents

All High Performance Liquid Chromatography (HPLC) standards, and ascorbic acid; 2,2-azinobis (3-ethylbenzthiazoline-6-sulphonic acid) (ABTS); 2,2-Diphenyl-1-picryhydrazyl (DPPH) radical were purchased from Sigma-Aldrich (St. Louis, Missouri, United States). However, solvents viz. ethanol, isopropanol and methanol were procured from HiMedia Laboratories Pvt. Ltd. (Mumbai, India). Used HPLC standards were of HPLC grade and other chemicals and solvents were of analytical grade.

### 2.3. Solid–liquid extraction

**Fig 1:**
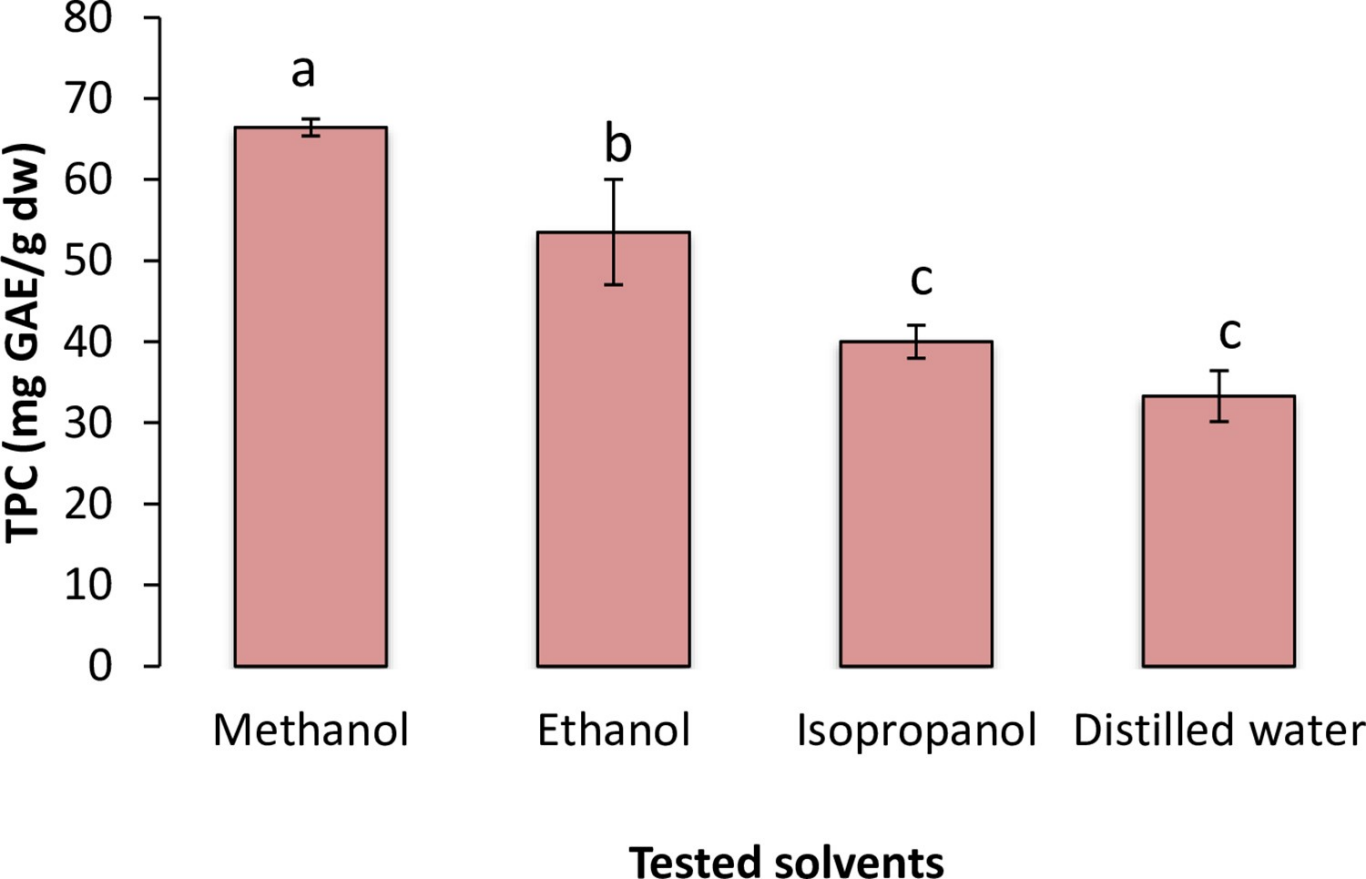
Solvent selection and response of different solvents on total polyphenolic content (TP). Bars capped with same letters are not significantly *(p < 0*.*05)* different to each other and separated using Duncan’s Multiple Range Test (DMRT).

Solvents of different polarities were examined for choosing appropriate solvent (Fig 1). Two gram leaf powder (sample) of *Q*. *semecarpifolia* was dissolved in 20 mL of 80% solvent (having reaming 20% distilled water) and kept at 60°C for heat assisted extraction in water-bath (Toshiba, India) for 60 min. Whatman paper (no 1) was used for phase separation. By considering the higher yield of total phenolic content (TP), best solvent was selected.

### 2.4. Experimental plan

The experimental plan followed a two-level multifactor design. Initially, a five factor [i.e., X_A_: Extraction temperature (°C), X_B_: Extraction time (min), X_C_: Solvent (methanol) concentration (%), X_D_: Solvent pH, X_E_: sample-to-solvent ratio (g/ml)] Plackett-Burman statistical design (PBD) involving 12 experimental runs considering only one dependent variable [Total Phenolic Content] was performed to select the influential factors (Table 1). In next step, most influencing three-factors [i.e., X_1_: Extraction temperature (°C), X_2_: Solvent concentration (%) and X_3_: Sample-to-solvent ratio (g/ml], were used in Box-Wilson Central Composite Design (CCD); linking 20 experiments considering five dependent variables [i.e. Total Phenolic Content, Total Flavonoid Content, Total Tannin Content, Total Antioxidant Activity (ABTS and DPPH)] (Table 2). Table 3 shows the operational conditions examined for individual dependent variable. PBD is mainly used to separate main contributing factors in extraction process and does not explain interactions between factors. In present study only those factors having confidence level >95% were considered. It was followed by Box-Behnken design (BBD) to screen process variables and their interactions on yield optimization of phytochemicals.

**Table 1:** Plackett-Burman design (PBD) with responses of the dependent variables to extraction conditions.

Independent variables						Dependent Variables
Experimental run	X_A_	X_B_	X_C_	X_D_	X_E_	TP(mg GAE/g dw)
1	30 (-1)	60(1)	60 (1)	6 (1)	1:15 (-1)	52.13
2	60 (1)	30(-1)	60 (1)	2.5 (-1)	1:15 (-1)	76.77
3	30 (-1)	30 (-1)	60 (1)	6 (1)	1:30 (1)	62.50
4	60 (1)	60 (1)	30 (-1)	6 (1)	1:30 (1)	67.42
5	60 (1)	60 (1)	30 (-1)	6 (1)	1:15 (-1)	58.88
6	60 (1)	60 (1)	60 (1)	2.5 (-1)	1:30 (1)	83.26
7	30 (-1)	30 (-1)	30 (-1)	6 (1)	1:30 (1)	53.62
8	60 (1)	30 (-1)	30 (-1)	2.5 (-1)	1:30 (1)	62.26
9	60 (1)	30 (-1)	60 (1)	6 (1)	1:15 (-1)	71.99
10	30 (-1)	60 (1)	60 (1)	2.5 (-1)	1:30 (1)	67.70
11	30 (-1)	60 (1)	30 (-1)	2.5 (-1)	1:15 (-1)	47.14
12	30 (-1)	30 (-1)	30 (-1)	2.5 (-1)	1:15 (-1)	47.14

X_A_ = Extraction temperature (°C), X_B_ = Extraction time (min), X_C_ = Solvent concentration (%), X_D_ = pH, X_E_ = Sample-to-solvent ratio (g/ml), TP = Total polyphenolic content

**Table 2:** Box-Wilson Central Composite Design (CCD) with responses of the dependent variables to extraction conditions.

Independent variables				Dependent Variables				
Experimental run	X_A_	X_C_	X_E_	TP(mg GAE/g dw)	TT(mg TAE/g dw)	TF(mg QE/g dw)	DPPH(mM AAE/g dw)	ABTS(mM AAE/g dw)
1	60(0)	70(0)	1:30(0)	64.66	67.54	27.89	25.44	1.78
2	40(-1)	50(-1)	1:40(+1)	46.27	73.63	22.69	39.71	1.23
3	60(0)	70(0)	1:20(-1)	48.74	44.29	18.65	18.46	1.26
4	60(0)	90(+1)	1:30(0)	42.76	70.76	52.51	28.22	1.35
5	40(-1)	90(+1)	1:20(-1)	32.78	41.84	37.82	19.37	1.02
6	80(+1)	50(-1)	1:20(-1)	53.73	41.58	16.73	20.23	1.27
7	60(0)	70(0)	1:30(0)	51.53	64.24	24.14	27.14	1.63
8	60(0)	70(0)	1:30(0)	64.52	59.57	26.25	27.59	1.82
9	40(-1)	90(+1)	1:40(+1)	31.77	76.31	46.41	38.58	0.94
10	60(0)	70(0)	1:30(0)	47.53	64.52	30.77	25.79	1.76
11	60(0)	70(0)	1:30(0)	49.58	59.88	30.10	26.35	1.77
12	80(+1)	90(+1)	1:40(+1)	68.92	88.81	42.56	37.31	2.20
13	60(0)	70(0)	1:40(+1)	64.96	84.30	30.38	40.42	1.96
14	40(-1)	70(0)	1:30(0)	40.46	60.80	24.52	29.10	1.36
15	80(+1)	70(0)	1:30(0)	91.27	61.52	31.45	31.19	1.89
16	60(0)	50(-1)	1:30(0)	47.49	58.38	25.48	25.54	1.63
17	80(+1)	90(+1)	1:20(-1)	65.42	42.43	33.33	17.86	1.26
18	60(0)	70(0)	1:30(0)	49.32	62.17	31.06	25.44	1.73
19	80(+1)	50(-1)	1:40(+1)	72.81	86.82	35.38	37.82	2.32
20	40(-1)	50(-1)	1:20(-1)	49.01	42.66	15.83	18.88	1.23

X_A_ = Extraction temperature (°C), X_C_ = Solvent concentration (%), X_E_ = Sample-to-solvent ratio (g/ml)

TP = Total polyphenolic content, TT = Total tannin content, TF = Total flavonoids content, DPPH = 2,2-diphenyl-1- picrylhydrazyl radical scavenging ability, ABTS = 2,2’-azino-bis (3-ethylbenzothiazoline-6-sulphonic acid) radical cation inhibition

**Table 3:** Analysis of variance (ANOVA) of the regression model from the Plackett-Burman Design (PBD) for major contribution to total phenol content.

Source	Sum of Squares	DF	Mean Square	*F* value	*P* value
Model	1364.45	5	272.89	19.79	0.0011**
X_A_	680.13	1	680.13	49.33	0.0004***
X_B_	0.42	1	0.42	0.03	0.8665
X_C_	505.62	1	505.62	36.67	0.0009***
X_D_	26.24	1	26.24	1.90	0.2169
X_E_	152.03	1	152.03	11.03	0.0160*
Residual	82.73	6	13.79		
Total	1447.18	11			

X_A_ = Extraction temperature (°C), X_B_ = Extraction time (min), X_C_ = Solvent concentration (%), X_D_ = pH, X_E_ = Sample-to-solvent ratio (g/ml).DF = degrees of freedom.

Level of significance

* p < 0.05

** p < 0.01

*** p < 0.001.

### 2.5. Analytical methods

#### 2.5.1. The total phenolic content (TP)

TP was calculated by following Folin-Ciocaltue’s method described by Singleton and Rossi [41] and the content was quantified as miligram gallic acid equivalent per gram of dry leaf sample (mg GAE/g dw).

#### 2.5.2. Total flavonoid content (TF)

The Aluminium chloride (AlCl_3_) method [42] was used for calculating total flavonoid content (TF) in *Quercus* leaf samples and quantified as miligram quercetin equivalent per of gram dry leaf sample (mg QE/g dw).

#### 2.5.3. Total tannin content (TT)

The Folin-Denis colorimetric method was used to measure total tannin content (TT) by following Ram and Mehrotra [43], and results were expressed as miligram tannic acid equivalent per gram of dry leaf sample (mg TAE/g dw).

#### 2.5.4. Total Antioxidant Activity (TAA)

The antioxidant activity in *Quercus* leaf samples was analyzed through ABTS and DPPH assays by following Pandey et al. [19] and results were presented as millimolar ascorbic acid equivalent per gram of dry leaf sample (mM AAE /g dw).

### 2.6. HPLC analysis

The phenolic profiles of extracts were analyzed through high performance liquid chromatography (HPLC) (Shimadzu LC-10AT, Japan) coupled with diode array detector (DAD-MZOA) and two LC-10AT HPLC pumps [44]. Phenolic compounds were quantified by using peak area and prepared standard curve of corresponding phenolic compound standard. Each quantification of phenolic compounds was repeated three times for each *Quercus* species and expressed as milligram per gram dry weight of leaf samples (mg/g dw).

### 2.7. Statistical analysis

All analytical experiments were repeated three times, except RSM analysis, and obtained results were analyzed through analysis of variance (ANOVA) using SPSS statistical package for Windows (IBM SPSS Statistics 20, Chicago, USA). The individual and interrelated influence of significant factors on the extraction yield were studied by plotting three dimensional (3D) response surface plots through Design-Expert^®^ version 12 software (Stat-Ease, Inc., MN, USA) and multiple regression analysis opted to analyze experimental data [45].

## 3. Result and discussion

### 3.1. Selection of solvents

Solvent type and solvent concentration are known to influence extraction yield and extraction of phytochemicals [7]. Four solvents were tested to determine the maximum TP content from *Quercus* leaves. Among all tested solvents (80% v/v), significantly (*p<0*.*05*) higher yield of TP (66.43 mgGAE/g dw) was obtained in methanol followed by ethanol (53.51 mgGAE/g dw), isopropanol (40.02 mgGAE/g dw) and distilled water (33.30 mgGAE/g dw) (Fig 1). Thus methanol was used for further experiments.

### 3.2. Selection of significant factor

**Fig 2:**
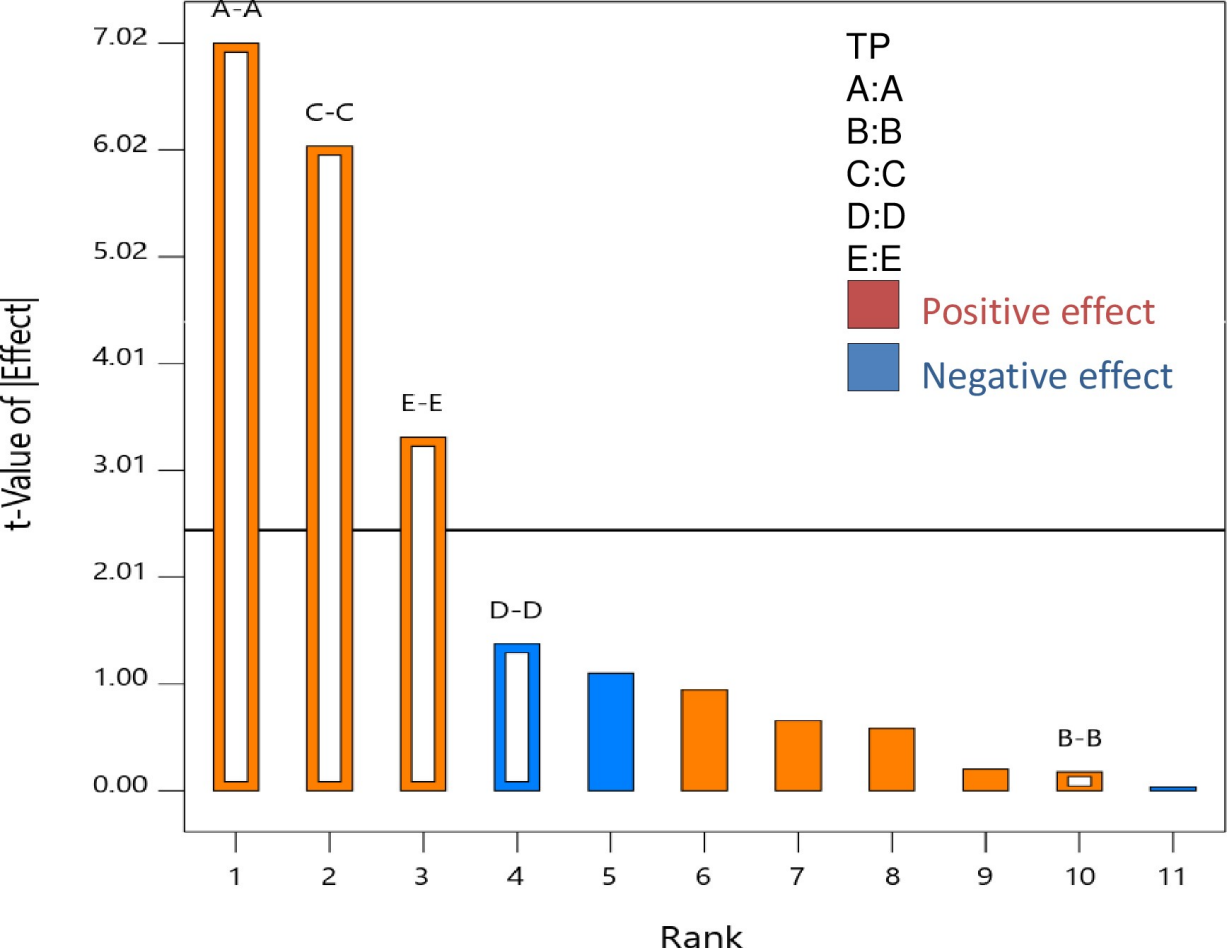
Pareto chart showing responses of the dependent variables to extraction conditions under Plackett-Burman design (PBD). A = Extraction temperature (°C), B = Extraction time (min), C = Solvent concentration (%), D = pH, E = Sample-to-solvent ratio (w/v), TP = Total polyphenolic content.

Plackett–Burman Design (PBD) was applied using five possible factors, namely, X_A_: extraction temperature (°C), X_B_: extraction time (min), X_C_: solvent concentration (%), X_D_: solvent pH, X_E_: sample-to-solvent ratio (g/ml) on TP concentration response. The analysis of variance (ANOVA) of the response data showed a significant effect of extraction temperature, solvent concentration, and sample-to-solvent ratio on TP response (Table 1). Overall, the PBD model was found significant *(p<0*.*01)* with F-value of 19.79 and showed good determination coefficient value (0.94). All the significant factors showed positive regression coefficient value which revealed that the TP response increased with increase in extraction temperature, solvent concentration and sample-to-solvent ratio. Among these, the influence of extraction temperature on TP response was the maximum followed by solvent concentration and sample-to-solvent ratio (Fig 2). The remaining factors which includes, solvent pH (2) and extraction time (45 min) were kept at a constant value for further experiments.

### 3.3. Model fitness

Box-Wilson central composite design (BW-CCD) model was applied on significant factors over 3 levels to determine the linear, interactive and quadratic effect of factors on polyphenolic contents and antioxidant activity. To best fit the model in determining the optimized condition, model fitness analysis was conducted. All response models showed model fitness with the significant *F-value*, which indicates the significant role of factor’s level in deterring the model variations. The coefficient of determination value was also found satisfactory for TP response, while for others it showed good R^2^ value (Table 4). The insignificant lack of fit value was recorded for all responses except for DPPH. For all response models at least one factor has shown a significant linear, interactive and quadratic effect. As such, for TP response, the linear effect of extraction temperature was found significantly *(p<0*.*001)* positive, while the solvent concentration showed significant *(p<0*.*05)* negative effect on TP. For TT response, a highly significant positive linear influence of extraction temperature *(p<0*.*05)* as well as sample-to-solvent ratio *(p<0*.*001)* was recorded. Moreover, a positive interactive effect between these factors has also affected TT response significantly *(p<0*.*05)*. Similarly, in case of TF, a highly significant *(p<0*.*001)* positive linear effect of solvent concentration and sample-to-solvent ratio was recorded. Also, solvent concentration was found to produce a highly significant *(p<0*.*001)* positive quadratic effect on TF. A significantly *(p<0*.*05)* negative effect of sample-to-solvent ratio and an interactive effect between extraction temperature and solvent concentration was recorded for TF.

**Table 4:** Regression coefficient (β), coefficient of determination (R^2^) and F-test value of the predicted second order polynomial models (CCD) for polyphenolics and antioxidant activities.

Regression Coefficients (β)					
	TP(mg GAE/g dw)	TT(mg TAE/g dw)	TF(mg QE/g dw)	DPPH(mM AAE/g dw)	ABTS(mM AAE/g dw)
Intercept, X_0_	55.42	63.32	28.86	27.04	1.72
Linear					
X_A_	15.19***	2.59*	1.22	-0.1228	0.315***
X_C_	-2.77	1.71	9.65***	-0.085	-0.092**
X_E_	3.5	19.71***	5.51***	9.91***	0.2617***
Quadratic					
X_A_^2^	9.08	-2.66	-1.63	1.98	-0.0518
X_C_^2^	-11.65*	0.7504	9.38***	-1.29	-0.1841**
X_E_^2^	0.0711	0.4773	-5.09*	1.27	-0.0624
Cross Product					
X_A_X_C_	4.82	0.12	-2.74*	-0.2806	0.0472
X_A_X_E_	3.29	3.27*	1.55	-0.375	0.2587***
X_C_X_E_	-1.73	0.5782	-0.9615	0.0306	-0.0222
R^2^	0.827098	0.973231	0.935638	0.972387	0.973269
*F value* (model)	5.32**	40.4***	16.15***	39.13***	40.46***
*F value* (lack of fit)	1.18	1.41	1.83	5.97*	2.33
*P value*	0.4296	0.3569	0.2612	0.0361	0.1879

X_A_ = Extraction temperature (°C), X_C_ = Solvent concentration (%), = X_E_ = Sample-to-solvent ratio (g/ml), TP = Total polyphenolic content, TT = Total tannin content, TF = Total flavonoids content, DPPH = 2,2-diphenyl-1- picrylhydrazyl radical scavenging ability, ABTS = 2,2’-azino-bis (3-ethylbenzothiazoline-6-sulphonic acid) radical cation inhibition; Level of significance

* p < 0.05

** p < 0.01

*** p < 0.001.

**Fig 3:**
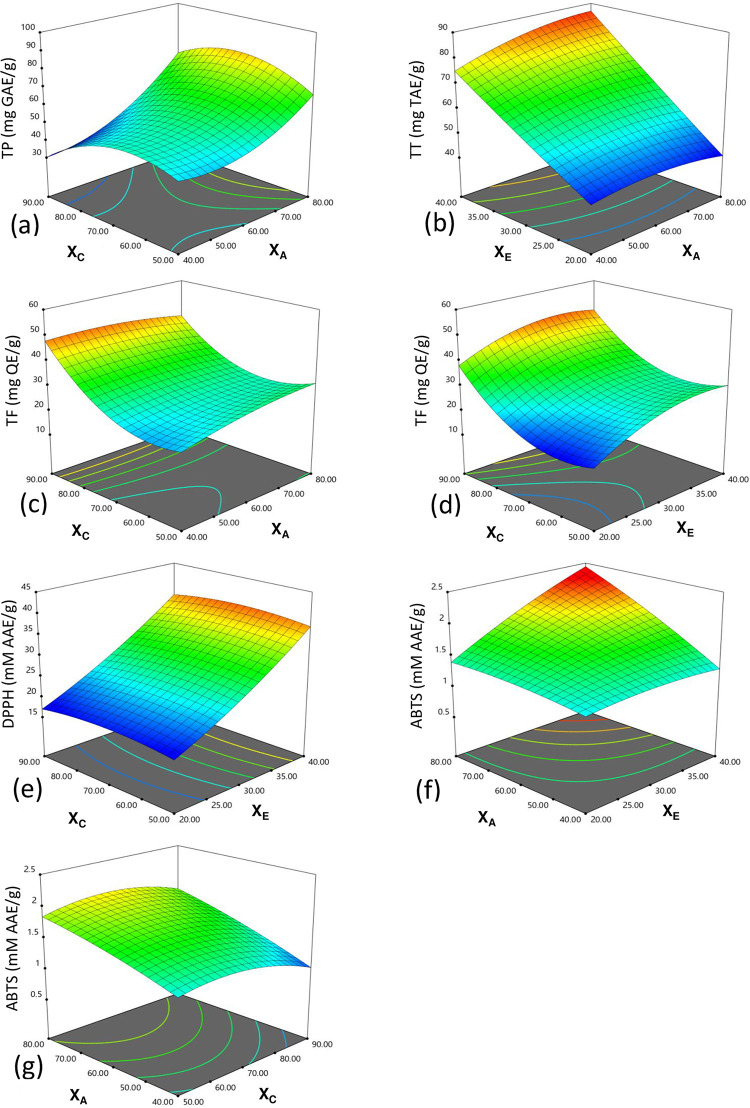
Response surface graphs showing the linear, quadratic, and interactive effect of different factors under Central Composite Design (CCD). X_A_ = Extraction temperature (°C), X_C_ = Solvent concentration (%), X_C_ = Sample-to-solvent ratio (g/ml), TP = Total polyphenolic content, TT = Total tannin content, TF = Total flavonoids content, DPPH = 2,2-diphenyl-1- picrylhydrazyl radical scavenging ability, ABTS = 2,2’-azino-bis (3-ethylbenzothiazoline-6-sulphonic acid) radical cation inhibition.

The antioxidant activity was analyzed using DPPH and ABTS *in vitro* assay. Both the assays were highly influenced by sample-to-solvent ratio and shown a significant *(p<0*.*001)* positive linear effect (Fig 3E and 3F). Individually, ABTS activity showed a highly significant *(p<0*.*001)* positive dependence on the extraction temperature. Also, a positive interactive effect of extraction temperature and solvent concentration was recorded for ABTS activity. However, significantly *(p<0*.*01)* negative linear and quadratic effects of solvent concentration on ABTS activity were observed.

All the significant linear, quadratic and interactive term fitted to the second order polynomial equation as-

YTP = 55.42 + 15.19X_A_—11.65X_C_^2^

YTT = 66.32 + 2.59X_A_ + 19.71X_E_ + 3.27X_A_X_C_

YTF = 28.86 + 9.65X_C_ + 5.51X_E_ + 9.38X_C_^2^–5.09X_E_^2^–2.74X_A_X_C_

YDPPH = 27.04 + 9.91X_E_

YABTS = 1.72 + 0.315X_A_—0.092X_C_ + 0.2617X_E_—0.1841X_C_^2^ + 0.2587X_A_X_E_

### 3.4. Effects of factors on the responses

#### 3.4.1. Effect of extraction temperature

Extraction temperature played a major role in polyphenolic contents as well as antioxidant activity from Oak leaves. Linear effect of extraction temperature significantly affected TP, TT and ABTS antioxidant activity. With an increase in extraction temperature from 40°C to 80°C, a highly significant *(p<0*.*001)* linear increase of TP content was recorded (Fig 3A). Similarly, a significant *(p<0*.*05)* increase in TT content was recorded with increasing temperature. However, the effect of extraction temperature was more on TP response compared to TT response (Fig 3B). Likewise, a highly significant *(p<0*.*001)* linear increase in ABTS activity was seen with increasing extraction temperature and sample-to-solvent ratio (Fig 3F). A highly significant *(p<0*.*001)* increase in ABTS activity was seen when both sample-to-solvent ratio and extraction temperature were raised simultaneously. Also, TT increased significantly *(p<0*.*05)* with the increase in extraction temperature along with sample-to-solvent ratio (Fig 3B). Though, increasing solvent concentration and extraction temperature, has a significant *(p<0*.*05)* negative effect on TF. Further, with decreasing extraction temperature and increase in solvent concentration, TF increased significantly (Fig 3C). The heat energy known to improve the effectiveness of extraction by distorting cellular structures, increasing the permeability of cell membrane and breaking-down of polyphenol-lipoprotein interactions, which leads to increased solubility and mass transfer of PAs into the solvent [46]. At high temperature the viscosity of extraction medium decreases, that helps solvents to enter the plant matrix leading to rapid kinetics [47]. Further, with the increase of solvent temperature surface tension decreases, that may increase wetting of the plant-particles, and resulting a higher extraction yield form softened plant tissue [48].

#### 3.4.2. Effect of solvent concentration

With an increase in solvent concentration from 50% to 90%, a highly significant *(p<0*.*001)* increase in TF concentration was recorded (Fig 3C). At higher solvent concentration, the TF increased profoundly as compared to the linear increase and showed highly significant *(p<0*.*001)* positive quadratic effect. However, ABTS activity was observed decreasing with increasing solvent concentration (Fig 3G). Similarly, with increase in solvent concentration at higher level, TP significantly *(p<0*.*05)* decreased. Also, with increase in extraction temperature the positive influence of solvent concentration on TF was found to be decreased and revealed a negative interactive effect (Fig 3C). Studies have indicated the efficacy of methanol over other organic solvents in phytochemical extraction [49, 50].

#### 3.4.3. Effect of sample-to-solvent ratio

A highly significant *(p<0*.*001)* positive linear effect of sample-to-solvent ratio was found on all responses except TP (Table 4 and Fig 3). Increasing sample-to-solvent ratio from 1:20 to 1:40 (w/v), a significant linear enhancement in TF, TT, ABTS and DPPH was recorded. It can be correlated to the hindrance of saturation in extraction medium by using higher volume of solvent [51, 52]. Also, with increasing extraction temperature and sample-to-solvent ratio, the TT and ABTS values were increased significantly. Optimized sample-to-solvent ratio is essential to develop equilibrium between high extraction costs and wastage of solvents and deterrence of saturation effects [53]. It is reported that some polyphenolic compounds possess complex structures that are soluble only in organic solvents and their combinations often with different proportions of water [7]. Therefore, increasing methanol concentration may enhance the extraction of soluble polyphenolic compounds from plant samples. Furthermore, being the high polarity solvent, methanol improves the release of bioactive compound and may serve as a green solvent for extraction of PAs [54]. According to the study by Capello et al. [50] on comprehensive framework for the environmental assessment of solvents, among tested 26 organic solvents in order to identify green solvents, methanol–water or ethanol–water mixtures were emerged environmentally benign compared to pure alcohol or propanol–water mixtures.

### 3.5. Optimized HAE condition and its validation

Based on the above experiments (section 3.1–3.4), the optimized heat assisted extraction (HAE) condition for *Q*. *semecarpifolia* was determined as: temperature (80°C), solvent concentration (87% methanol), sample-to-solvent ratio (1: 40), solvent pH (2), and extraction time (45 min). The optimized condition was further tested on five *Quercus* species i.e. *Q*. *floribunda*, *Q*. *franchetii*, *Q*. *glauca*, *Q*. *oblongata*, and *Q*. *serrata*. The TP was recorded maximum (77.34 mg GAE/g dw) in *Q*. *semecarpifolia* and minimum (63.01 mg GAE/g dw) in *Q*. *serrata*. However, TT (94.19 mg TAE/g dw) and TF (87.05 mg QE/g dw) content were observed maximum in *Q*. *serrata* and minimum TT (80.10 mg TAE/g dw) in *Q*. *franchetii* and TF (27.24 mg QE/g dw) in *Q*. *glauca* respectively (Table 5). All values were found close to the model’s predicted value which indicates that the model is well-fit for the extraction of polyphenolic compounds from *Quercus* leaves under optimal HAE condition and the used RSM models are good for predicting the optimal extraction condition.

**Table 5:** Comparative phytochemical profile of *six Quercus species* under optimal extraction condition.

Phytochemical screening	*Quercus* species					
	*Q*. *glauca*	*Q*. *oblongata*	*Q*. *floribunda*	*Q*. *franchetii*	*Q*. *semecarpifolia*	*Q*. *serrata*
***Phytochemicals and antioxidant activity***						
TP(mg GAE/g dw) [*pv* 78.44]	65.04	74.85	67.97	70.70	77.34	94.19
TT(mg TAE/g dw) [*pv* 89.29]	84.72	86.42	90.23	80.10	91.45	87.05
TF(mg QE/g dw) [*pv* 42.23]	37.24	27.24	38.97	31.67	44.42	33.10
DPPH(mM AAE/g dw) [*pv* 38.49]	42.22	39.09	37.62	42.51	35.35	2.18
ABTS(mM AAE/g dw) [*pv* 2.25]	2.16	2.14	2.15	2.14	2.10	63.01
***Phenolic compounds***	***Concentration (mg/g dw)***					
3-Hydroxy Benzoic acid	3.69±0.04	1.14±0.31	2.24±0.12	1.96±0.15	-	1.95±0.23
Caffeic acid	-	-	-	-	0.09±0.00	0.03±0.00
Catechin	-	-	37.60±0.65	2.20±0.28	28.31±0.00	19.53±0.58
Chlorogenic acid	5.22±0.08	6.21±0.12	-	1.30±0.17	-	-
Ellagic acid	-	-	-	0.10±0.00	-	-
Ferulic acid	-	0.23±0.00	-	0.15±0.02	-	0.129±0.00
Gallic acid	23.45±0.19	28.28±0.32	14.81±0.18	21.55±1.41	16.53±0.24	17.28±0.06
Rutin	-	-	-	-	3.51±0.03	3.52±0.03
Trans cinnamic acid	0.04±0.00	0.10±0.00	-	0.01±0.00	-	-
Vanillic acid	0.44±0.01	0.60±0.05	-	0.25±0.01	0.16±0.01	0.44±0.02
m-coumaric acid	0.25±0.01	-	-	-	-	-
p-coumaric acid	-	-	-	-	0.10±0.01	0.11±0.00

TP = Total polyphenolic content, TT = Total tannin content, TF = Total flavonoids content, DPPH = 2,2-diphenyl-1- picrylhydrazyl radical scavenging ability, ABTS = 2,2’-azino-bis (3-ethylbenzothiazoline-6-sulphonic acid) radical cation inhibition

*Pv* = model’s predicted value; - = not detected

### 3.6. Polyphenolic screening

Under optimum HAE condition, the HPLC analysis revealed presence of 12 antioxidant polyphenolic compounds (caffeic acid, catechin hydrate, chlorogenic acid, ellagic acid, ferulic acid, gallic acid, rutin hydrate, trans cinnamic acid, *m*-coumaric acid, *p*-coumaric acid, 3-hydroxybenzoic acid and vanillic acid) in the leaf extracts of total screened (six) *Quercus* species, described in section 3.5 (Table 5). The polyphenolic compound composition and concentration varied in target species. *Q*. *franchetii* and *Q*. *serrata* were among the highest (8 in each) polyphenolic compound containing species followed by *Q*. *glauca*, *Q*. *oblongata*, *Q*. *semecarpifolia* (6 each) and *Q*. *floribunda* (3). Among the detected compounds, catechin was found in highest concentrations in *Q*. *floribunda* (37.60 mg/g leaf dw), *Q*. *semecarpifolia* (28.31 mg/g leaf dw) and *Q*. *serrata* (19.53 mg/g leaf dw) respectively. Followed by gallic acid, and it was detected maximum in *Q*. *oblongata* (28.276 mg/g leaf dw), *Q*. *glauca* (23.45mg/g leaf dw), and *Q*. *franchetii* (21.55 mg/g leaf dw) respectively (Table 5). The quantified polyphenolic compounds from six Himalayan *Quercus* species such as rutin, p-coumaric acid, catechin, gallic acid, 3-hydroxybenzoic acid, vanillic acid, caffeic acid, ferulic acid, ellagic acid, m-cinnamic acid, p-cinnamic acid and chlorogenic acid have gained significant attention due to their multiple biological activities including antioxidant, antimicrobial, anticancer, antidiabetic, anti-inflammatory, antihepatotoxic, anticholestatic, antihepatocarcinogenic antisteatosic, free radical scavenging, cytotoxic, gastric protective and inhibition of HIV replication [55–58]. The highest recorded phenolic compounds in the current studied *Quercus* species *i*.*e*. catechin and gallic acid are known to present in many dietary products, plants, fruits, etc. and clinical studies have shown the beneficial effects of catechin and gallic acid due their antioxidant action [59]. Catechin plays a significant role in several molecular mechanisms such as angiogenesis, degradation of extracellular matrix, regulation of cell death, and multidrug resistance in cancer and associated disorders [59]. Reports indicate that the PAs in studied Himalayan *Quercus* species are comparable to various medicinal plants of Himalayan region studied in same laboratory conditions such as 12 PAs in [*Rheum moorcroftianum* under ultrasonic assisted extraction [19]; 6, 7 PAs in *B*. *jaeschkeana* and *B*. *asiatica* respectively under microwave assisted extraction [43], 13 PAs in different *in vitro* growing stages of *Origanum vulgare* under heat assisted extraction [44]; 10 and 9 PAs under microwave assisted extraction and ultrasonic assisted extraction respectively in *Berberis jaeschkeana* [60]. Furthermore, this is the first report on extraction optimization of twelve antioxidant polyphenolic compounds in a single extraction process from Himalayan *Quercus* species. Considering the significant extraction yield of polyphenolic compounds such as gallic acid, catechin, chlorogenic acid, etc. this optimized HAE condition can be extended to the industrial purpose.

## 4. Conclusion

Among six *Quercus* species examined in the present study, *Q*. *semecacrpifolia* and *Q*. *serrata* exhibited significantly higher levels of polyphenols under the optimized extraction condition i.e. extraction temperature (80°C), solvent concentration of (87%; 0.2N methanol), sample-to-solvent ratio (1: 40), solvent pH (2), and extraction time (45 min). Gallic acid was observed in all the tested *Quercus* species however, highest catechin concentration was observed for *Q*. *floribunda*. *Quercus glauca* and *Q*. *franchetii* had significantly stronger DPPH radical scavenging activity or reducing power compared with that of the other tested species. No significant difference was observed in ABTS radical scavenging activity of tested six *Quercus* species. This study indicates that all the tested *Quercus* species of central Himalaya have a high utilization potential because of their high phenolic and flavonoid contents as well as strong antioxidant nature. Further, presence of significant catechin concentration in leaves of these species indicates use of *Quercus* species especially *Q*. *floribunda*, *Q*. *semecarpifolia* and *Q*. *serrata* in nutraceutical interventions and the extract after evaporating the solvent can be used in many nutrient formulations like beverages, dietary products etc. Use of *Quercus* species in pharmaceuticals and nutraceuticals may be helpful in reducing the extraction pressure from the threatened medicinal plants of Himalaya.

## Supporting information

S1 File (DOCX)

## References

[1] Pandey K. B., Rizvi S. I. Plant polyphenols as dietary antioxidants in human health and disease. *Oxidative medicine and cellular longevity*, 2009; 2(5), 270–278. doi: 10.4161/oxim.2.5.9498 20716914 PMC2835915

[2] Saad B., Sing Y. Y., Nawi M. A., Hashim N. H., Ali A. S. M., Saleh M. I., et al. Determination of synthetic phenolic antioxidants in food items using reversed-phase HPLC. Food Chemistry, 2007; 105, 389–394. 10.1016/j.foodchem.2006.12.025

[3] Shahidi F., Ambigaipalan P. Phenolics and polyphenolics in foods, beverages and spices: Antioxidant activity and health effects–A review. Journal of functional foods, 2015; 18, 820–897. 10.1016/j.jff.2015.06.018

[4] McClements D. J., Decker E. A. Lipids. In Damodaran S, Parkin K. L, & Fennema O. R (Eds.), Fennema’s food chemistry, 2008; (pp. 156–212). NewYork: CRC Press. doi: 10.1016/j.foodchem.2008.01.057

[5] Dolatabadi J. E. N., Kashanian S. A review on DNA interaction with synthetic phenolic food additives. Food Research International, 2010; 43, 1223–1230. 10.1016/j.foodres.2010.03.026

[6] Berdahl D., Nahas R. I., Barren J. P. Synthetic and natural additives in food stabilization: Current applications and future research. In Decker E. A, Elias R. J., & McClements D. J. (Eds.), Oxidation in foods and beverages and antioxidant applications 2010; (pp. 272–320). Oxford, UK: Woodhead Publishing. 10.1533/9780857090447.2.272

[7] Dai J., Mumper R. J. Plant phenolics: extraction, analysis and their antioxidant and anticancer properties. *Molecules*, 2010; 15(10), 7313–7352. doi: 10.3390/molecules15107313 20966876 PMC6259146

[8] Jain S., Jain A., Jain S., Malviya N., Jain V., Kumar D. Estimation of total phenolic, tannins, and flavonoid contents and antioxidant activity of *Cedrus deodara* heart wood extracts. *Egyptian Pharmaceutical Journal*, 2015; 14(1), 10. doi: 10.4103/1687-4315.154690

[9] Ghasemi K., Ghasemi Y., Ebrahimzadeh M. A. Antioxidant activity, phenol and flavonoid contents of 13 citrus species peels and tissues. *Pak J Pharm Sci*, 2009; 22(3), 277–281. 19553174

[10] Pokorný J. Are natural antioxidants better–and safer–than synthetic antioxidants?. *European Journal of Lipid Science and Technology*, 2007; 109(6), 629–642. doi: 10.1002/ejlt.200700064

[11] Craft B. D., Kerrihard A. L., Amarowicz R., Pegg R. B. Phenol‐based antioxidants and the in vitro methods used for their assessment. *Comprehensive Reviews in Food Science and Food Safety*, 2012; 11(2), 148–173. doi: 10.1111/j.1541-4337.2011.00173.x

[12] Colomer R., Sarrats A., Lupu R., Puig T. Natural polyphenols 412 and their synthetic analogs as emerging anticancer agents. Current drug targets, 2017; 18(2), 147–159. doi: 10.2174/1389450117666160112113930 26758667

[13] Wanyo P., Meeso N., Siriamornpun S. Effects of different treatments on the antioxidant properties and phenolic compounds of rice bran and rice husk. *Food chemistry*, 2014; 157, 457–463. doi: 10.1016/j.foodchem.2014.02.061 24679804

[14] Hamid M. R. Y., Ab Ghani M. H., Ahmad S. Effect of antioxidants and fire retardants as mineral fillers on the physical and mechanical properties of high loading hybrid biocomposites reinforced with rice husks and sawdust. *Industrial Crops and Products*, 2012; 40, 96–102. doi: 10.1016/j.indcrop.2012.02.019

[15] Nixon K. C. Infrageneric classification of *Quercus* (Fagaceae) and typification of sectional names. In *Annales des sciences forestières* (Vol. 50, No. Supplement, pp. 25s–34s), 1993; EDP Sciences. doi: 10.1051/forest:19930701

[16] Zillich O. V., Schweiggert‐Weisz U., Eisner P., Kerscher M. Polyphenols as active ingredients for cosmetic products. *International journal of cosmetic science*, 2015; 37(5), 455–464. doi: 10.1111/ics.12218 25712493

[17] Şöhretoğlu D., Sabuncuoğlu S., Harput Ü. Ş. Evaluation of antioxidative, protective effect against H_2_O_2_ induced cytotoxicity, and cytotoxic activities of three different *Quercus* species. *Food and chemical toxicology*, 2012; 50(2), 141–146. doi: 10.1016/j.fct.2011.10.061 22067294

[18] Khennouf S., Benabdallah H., Gharzouli K., Amira S., Ito H., Kim T. H. et al. Effect of tannins from *Quercus suber* and *Quercus coccifera* leaves on ethanol-induced gastric lesions in mice. *Journal of agricultural and food chemistry*, 2003; 51(5), 1469–1473. doi: 10.1021/jf020808y 12590500

[19] Pandey A., Belwal T., Sekar K. C., Bhatt I. D., Rawal R. S. Optimization of ultrasonic-assisted extraction (UAE) of phenolics and antioxidant compounds from rhizomes of *Rheum moorcroftianum* using response surface methodology (RSM). *Industrial Crops and Products*, 2018a; 119, 218–225. doi: 10.1016/j.indcrop.2018.04.019

[20] Pandey A., Sekar K. C., Tamta S., Rawal R. S. Assessment of phytochemicals, antioxidant and antimutagenic activity in micropropagated plants of *Quercus serrata*, a high value tree species of Himalaya. *Plant Biosystems-An International Journal Dealing with all Aspects of Plant Biology*, 2018b; 152(5), 929–936. doi: 10.1080/11263504.2017.1395372

[21] Kim J. J., Ghimire B. K., Shin H. C., Lee K. J., Song K. S., Chung Y. S., et al. Comparison of phenolic compounds content in indeciduous *Quercus* species. *Journal of Medicinal Plants Research*, 2012; 6(39), 5228–5239. doi: 10.5897/JMPR12.135

[22] Sánchez-Burgos J. A., Ramírez-Mares M. V., Larrosa M. M., Gallegos-Infante J. A., González-Laredo R. F., Medina-Torres L., et al. Antioxidant, antimicrobial, antitopoisomerase and gastroprotective effect of herbal infusions from four Quercus species. *Industrial Crops and Products*, 2013; 42, 57–62. doi: 10.1016/j.indcrop.2012.05.017

[23] Andrenšek S., Simonovska B., Vovk I., Fyhrquist P., Vuorela H., Vuorela P. Antimicrobial and antioxidative enrichment of oak (Quercus robur) bark by rotation planar extraction using ExtraChrom®. *International Journal of Food Microbiology*, 2004; 92(2), 181–187. doi: 10.1016/j.ijfoodmicro.2003.09.009 15109795

[24] Luthria D. L. Influence of experimental conditions on the extraction of phenolic compounds from parsley (Petroselinum crispum) flakes using a pressurized liquid extractor. *Food Chemistry*, 2008; 107(2), 745–752. doi: 10.1016/j.foodchem.2007.08.074

[25] Yilmaz F. M., Karaaslan M., Vardin H. Optimization of extraction parameters on the isolation of phenolic compounds from sour cherry (Prunus cerasus L.) pomace. *Journal of food science and technology*, 2015; 52(5), 2851–2859. doi: 10.1007/s13197-014-1345-3 25892783 PMC4397315

[26] Gong L., Huang L., Zhang Y. Effect of steam explosion treatment on barley bran phenolic compounds and antioxidant capacity. *Journal of agricultural and food chemistry*, 2012; 60(29), 7177–7184. doi: 10.1021/jf301599a 22708804

[27] Ahajji A., Diouf P. N., Aloui F., Elbakali I., Perrin D., Merlin A., et al. Influence of heat treatment on antioxidant properties and colour stability of beech and spruce wood and their extractives. *Wood Science and Technology*, 2009; 43(1–2), 69. doi: 10.1007/s00226-008-0208-3

[28] Lee S. C., Kim J. H., Jeong S. M., Kim D. R., Ha J. U., Nam K. C., et al. Effect of far-infrared radiation on the antioxidant activity of rice hulls. *Journal of agricultural and food chemistry*, 2003; 51(15), 4400–4403. doi: 10.1021/jf0300285 12848517

[29] Duh P. D., Yen G. C., Yen W. J., Chang L. W. Antioxidant effects of water extracts from barley (Hordeum vulgare L.) prepared under different roasting temperatures. *Journal of agricultural and food chemistry*, 2001; 49(3), 1455–1463. doi: 10.1021/jf000882l 11312880

[30] Majeed M., Hussain A. I., Chatha S. A., Khosa M. K., Kamal G. M., Kamal M. A., et al. Optimization protocol for the extraction of antioxidant components from *Origanum vulgare* leaves using response surface methodology. *Saudi Journal of Biological Sciences*, 2016; 23(3), 389–396. doi: 10.1016/j.sjbs.2015.04.010 27081365 PMC4818334

[31] Silva E. M., Rogez H., Larondelle Y. Optimization of extraction of phenolics from *Inga edulis* leaves using response surface methodology. *Separation and Purification Technology*, 2007; 55(3), 381–387. doi: 10.1016/j.seppur.2007.01.008

[32] De Castro M. L., Garcıa-Ayuso L. E. Soxhlet extraction of solid materials: an outdated technique with a promising innovative future. *Analytica chimica acta*, 1998; 369(1–2), 1–10. doi: 10.1016/S0003-2670(98)00233-5

[33] Barba F. J., Grimi N., Vorobiev E. Evaluating the potential of cell disruption technologies for green selective extraction of antioxidant compounds from *Stevia rebaudiana* Bertoni leaves. *Journal of Food Engineering*, 2015; 149, 222–228. doi: 10.1016/j.jfoodeng.2014.10.028

[34] Deng Q., Zhou X., Chen H. Optimization of enzyme assisted extraction of Fructus Mori polysaccharides and its activities on antioxidant and alcohol dehydrogenase. *Carbohydrate polymers*, 2014; 111, 775–782. doi: 10.1016/j.carbpol.2014.05.018 25037415

[35] Lee J. W., Mo E. J., Choi J. E., Jo Y. H., Jang H., Jeong J. Y., et al. Effect of Korean Red Ginseng extraction conditions on antioxidant activity, extraction yield, and ginsenoside Rg1 and phenolic content: optimization using response surface methodology. Journal of ginseng research, 2016; 40(3), 229–236. doi: 10.1016/j.jgr.2015.08.001 27616898 PMC5005304

[36] Nobre C. P., Raffin F. N., Moura T.F. Standardization of extracts from Momordica charantia L. (Cucurbitaceae) by total flavonoids content determination. *Acta Farmacéutica Bonaerense*, 2005; 24(4): 526–566.

[37] Tan M. C., Tan C. P., Ho C. W. Effects of extraction solvent system, time and temperatureon total phenolic content of henna (Lawsonia inermis) stems. *International Food Research Journal*, 2013; 20(6), 3117.

[38] Belwal T., Pandey A., Bhatt I.D., Rawal R.S. Optimized microwave assisted extraction (MAE) of alkaloids and polyphenols from Berberis roots using multiple-component analysis. *Scientific Reports*, 2020; 10, 917. doi: 10.1038/s41598-020-57585-8 31969583 PMC6976575

[39] Belwal T., Dhyani P., Bhatt I. D., Rawal R. S., Pande V. Optimization extraction conditions for improving phenolic content and antioxidant activity in Berberis asiatica fruits using response surface methodology (RSM). *Food Chemistry*, 2016; 207, 115–124. doi: 10.1016/j.foodchem.2016.03.081 27080887

[40] Vázquez G., Santos J., Freire M. S., Antorrena G., González-Álvarez J. Extraction of antioxidants from eucalyptus (Eucalyptus globulus) bark. *Wood science and technology*, 2012; 46(1–3), 443–457. doi: 10.1007/s00226-011-0418-y

[41] Singleton V. L., Rossi J. A. Colorimetry of total phenolics with phosphomolybdic-phosphotungstic acid reagents. *American journal of Enology and Viticulture*, 1965; 16(3), 144–158.

[42] Chang C. C., Yang M. H., Wen H. M., Chern J. C. Estimation of total flavonoid content in propolis by two complementary colorimetric methods. *Journal of food and drug analysis*, 2002; 10(3). ISSN: 1021-9498

[43] Ram P. R., Mehrotra B. N. Compendium of Indian medicinal plants, (Drug Research Preparative: A CDRI Series), Vol. 2, Central Drug Research Institute, Lucknow and Publications and Information Directorate, New Delhi, 1993.

[44] Pandey A., Belwal T., Tamta S., Bhatt I. D., Rawal R. S. Phenolic compounds, antioxidant capacity and antimutagenic activity in different growth stages of *in vitro* raised plants of *Origanum vulgare* L. *Molecular Biology Reports*, 2019; 46(2), 2231–2241. doi: 10.1007/s11033-019-04678-x 30756335

[45] Bourtoom T., Chinnan M. S., Jantawat P., Sanguandeekul R. Recovery and characterization of proteins precipitated from surimi wash-water. *LWT-Food Science and Technology*, 2009; 42(2), 599–605. doi: 10.1016/j.lwt.2008.09.001

[46] Mustafa A., Turner C. Pressurized liquid extraction as a green approach in food and herbal plants extraction: A review. *Analytica chimica acta*, 2011; 703(1), 8–18. doi: 10.1016/j.aca.2011.07.018 21843670

[47] Miron T. L., Plaza M., Bahrim G., Ibáñez E., Herrero M. Chemical composition of bioactive pressurized extracts of Romanian aromatic plants. *Journal of Chromatography A*, 2011; 1218(30), 4918–4927. doi: 10.1016/j.chroma.2010.11.055 21163488

[48] Vergara-Salinas J. R., Pérez-Jiménez J., Torres J. L., Agosin E., Pérez-Correa J. R. Effects of temperature and time on polyphenolic content and antioxidant activity in the pressurized hot water extraction of deodorized thyme (Thymus vulgaris). *Journal of agricultural and food chemistry*, 2012; 60(44), 10920–10929. doi: 10.1021/jf3027759 23075096

[49] Dhawan D., Gupta J. Comparison of Different Solvents for Phytochemical Extraction Potential from Datura metel Plant Leaves. *International Journal of Biological Chemistry*, 2017; 11: 17–22.

[50] Capello C., Fischer U., Hungerbühler K. What is a green solvent? A comprehensive framework for the environmental assessment of solvents. Green Chemistry, 2007; 9(9), 927–934. 10.1039/B617536H

[51] Ćujić N., Šavikin K., Janković T., Pljevljakušić D., Zdunić G., Ibrić S. Optimization of polyphenols extraction from dried chokeberry using maceration as traditional technique. *Food Chemistry*, 2016; 194, 135–142. doi: 10.1016/j.foodchem.2015.08.008 26471536

[52] Bucić-Kojić A., Planinić M., Tomas S., Bilić M., Velić D. Study of solid–liquid extraction kinetics of total polyphenols from grape seeds. *Journal of Food Engineering*, 2007; 81(1), 236–242. doi: 10.1016/j.jfoodeng.2006.10.027

[53] Pinelo M., Arnous A., Meyer A. S. Upgrading of grape skins: Significance of plant cell-wall structural components and extraction techniques for phenol release. *Trends in Food Science &* *Technology*, 2006; 17(11), 579–590. 10.1016/j.tifs.2006.05.003

[54] Bahtiar A., and Annisa R. Effects of dayak onion bulbs (Eleutherine bulbosa (Mill.) urb) on bone development of the hipoestrogen model rat. *Pharmacognosy Journal*, 2018; 10(2), 299–303. 10.5530/pj.2018.2.52

[55] Zduńska K., Dana A., Kolodziejczak A., Rotsztejn H. Antioxidant properties of ferulic acid and its possible application. *Skin pharmacology and physiology*, 2018; 31(6), 332–336. doi: 10.1159/000491755 30235459

[56] Yilmaz S., Sova M., Ergün S. Antimicrobial activity of trans‐cinnamic acid and commonly used antibiotics against important fish pathogens and nonpathogenic isolates. *Journal of Applied Microbiology*, 2018; 125(6), 1714–1727. doi: 10.1111/jam.14097 30179290

[57] García-Niño W. R., Zazueta C. Ellagic acid: pharmacological activities and molecular mechanisms involved in liver protection. *Pharmacological Research*, 2015; 97, 84–103. doi: 10.1016/j.phrs.2015.04.008 25941011

[58] Dhalwal K., Shinde V. M., Biradar Y. S., Mahadik K. R. Simultaneous quantification of bergenin, catechin, and gallic acid from *Bergenia ciliata* and *Bergenia ligulata* by using thin-layer chromatography. *Journal of food composition and analysis*, 2008; 21(6), 496–500. doi: 10.1016/j.jfca.2008.02.008

[59] Zanwar A. A., Badole S. L., Shende P. S., Hegde M. V., Bodhankar S. L. Antioxidant role of catechin in health and disease. In Polyphenols in human health and disease (pp. 267–271), 2014; Academic Press. doi: 10.1016/B978-0-12-398456-2.00021-9

[60] Belwal T., Giri L., Bhatt I. D., Rawal R. S., Pande V. An improved method for extraction of nutraceutically important polyphenolics from *Berberis jaeschkeana* CK Schneid. fruits. Food chemistry, 2017; 230, 657–666. doi: 10.1016/j.foodchem.2017.03.086 28407963

