# Supplementary material for: Optimized extraction of polyphenolic antioxidants from the leaves of Himalayan Oak species
Source: PLoS One. 2021 Nov 3;16(11):e0259350. doi: 10.1371/journal.pone.0259350 (PMC8565745; doi:10.1371/journal.pone.0259350)
Supplement: S1 File — (DOCX) [file pone.0259350.s001.docx]

**Methodology**

Fresh leaves were weighted using analytical weighing balance (Sartorius, India, Pvt. Ltd) and left for drying at room temperature. After complete drying (constant weight achieved in successive weighing), the dry weight of leaves was recorded. The moisture content (%) in leaves was calculated using following formula:

M_c_ = [(W_1_-W_2_)/W_1_] x 100

Where, M_c_: moisture content; W_1_: fresh weight of leaf; W_2_: dry weight of leaf.

S1 Table: Moisture content and leaf size of target Quercus species of Uttarakhand, West Himalaya

| Quercus species | Moisture Content (%) | Leaf Width (cm) | Leaf Length (cm) |
| --- | --- | --- | --- |
| *Q. glauca* | 42.69±0.55bc | 4.68±0.29ab | 15.80±0.64b |
| *Q. oblongata* | 49.67±1.61cd | 5.42±0.19bc | 15.60±0.70b |
| *Q. floribunda* | 34.69±4.63a | 3.84±0.10a | 09.02±0.25a |
| *Q. franchetii* | 47.21±1.95bcd | 7.78±0.46d | 18.20±1.40b |
| *Q. semecarpifolia* | 53.41±1.52d | 5.86±0.28c | 11.36±0.46a |
| *Q. serrata* | 41.56±2.40ab | 6.92±0.23d | 16.70±0.20b |

Values± SE in a column followed by same letters are not significantly (p<0.05) different and separated by Duncan’s multiple range test (DMRT).

**Description**

The leaf size of target species measured minimum (length: 09.02±0.25cm; width: 3.84±0.10 cm) for *Q. floribunda* and maximum (length: 18.20±1.40 cm; width: 7.78±0.46 cm) for *Q. franchetii* respectively. However, the moisture content varied significantly (p<0.05) among the leaves of tested *Quercus* species and ranged from 34.69±4.63 % in *Q. floribunda* to 49.67±1.61% in *Q. oblongata* (S1 Table).
